# Structural constraints prevent a bat norovirus from binding to histo-blood group antigen co-factors

**DOI:** 10.1128/jvi.00995-25

**Published:** 2025-08-29

**Authors:** Dayna L. Holroyd, John B. Bruning, Mark von Itzstein, Grant S. Hansman

**Affiliations:** 1 Institute for Photonics and Advanced Sensing (IPAS), School of Biological Sciences, The University of Adelaide, Adelaide, South Australia, Australia; 2 Institute for Biomedicine and Glycomics (IBG), Griffith University, Southport, Queensland, Australia; St. Jude Children's Research Hospital, Memphis, Tennessee, USA

**Keywords:** norovirus, X-ray crystallography, HBGA, co-factors

## LETTER

Noroviruses infect a plethora of animal species, including humans, dogs, and bats. While most research focuses on GI and GII human noroviruses, increasing attention is being directed toward animal noroviruses as possible viral reservoirs. One such group is GX bat noroviruses (1–3), which remain poorly understood but are gaining interest due to bats’ roles as reservoirs for zoonotic viruses. Indeed, a recent study showed that bat noroviruses bind histo-blood group antigens (HBGAs) (1), which are co-factors for human noroviruses (4, 5).

In this study, we observe novel structural factors on a GX bat norovirus (NPIH26) capsid that restrict HBGA binding and demonstrate that an emerging genotype, GII.27 binds an HBGA at a common pocket using X-ray crystallography.

**Fig 1:**
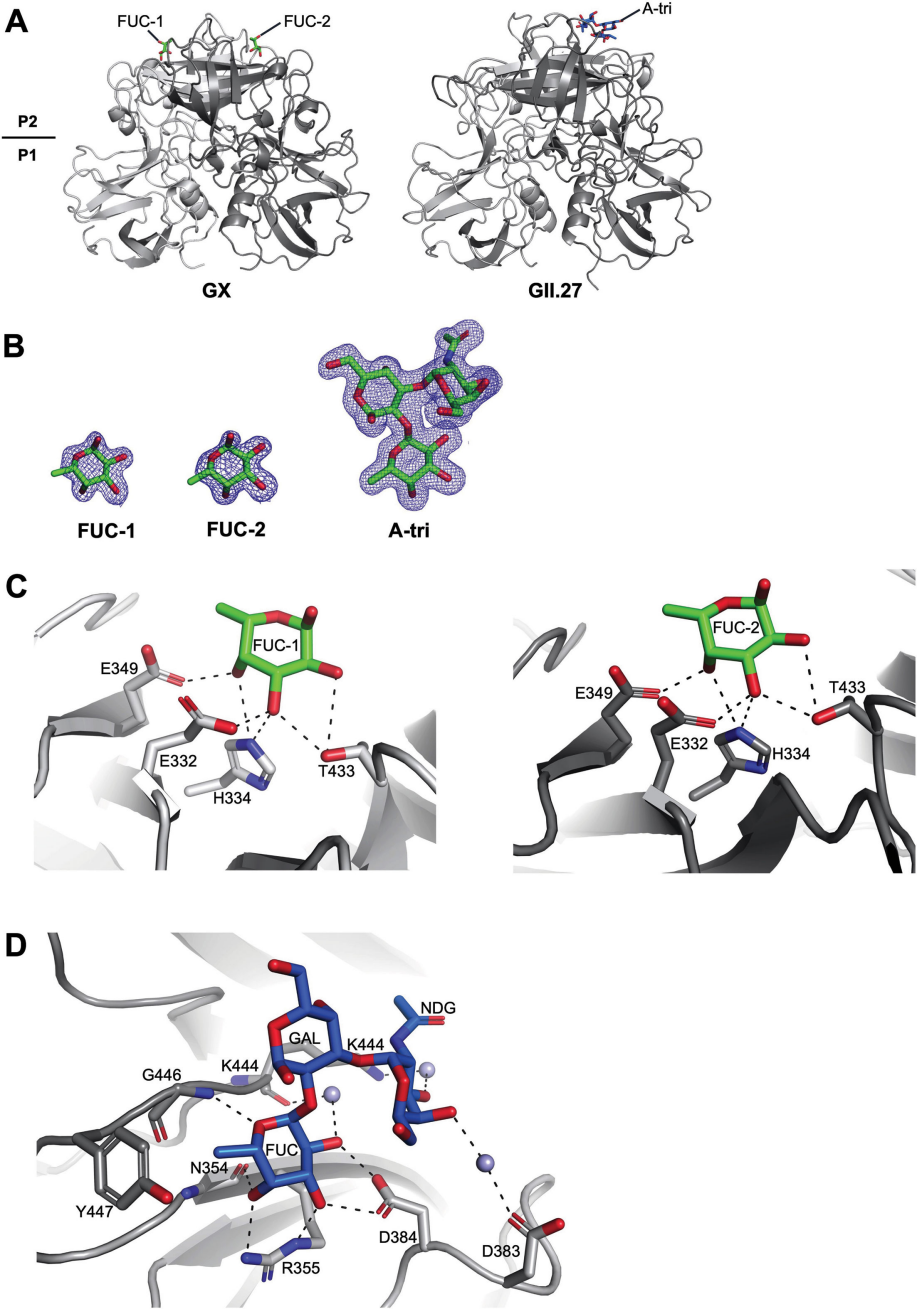
X-ray crystal structure of the GX P domain fucose complex and the GII.27 P domain A-trisaccharide complex. GX and GII.27 P domain proteins were co-crystallized with 30–60 molar excess of fucose and A-trisaccharide, respectively. (**A**) Two fucose molecules (termed FUC-1 and FUC-2) bound to the top of the GX P domain, whereas one A-trisaccharide molecule [α-L-fucose-(1-2)-α-D-galactose-(3-1)-2-*N*-acetyl-α-D-galactosamine] bound on the top of the GII.27 P domain. (**B**) The fucose and A-trisaccharide molecules were clearly visible after molecular replacement (and were only added during the final refinement to reduce biases). Representative simulated annealing *2Fo-Fc* composite omit map (blue) contoured at 1.0 sigma for the fucose moieties (FUC-1 and FUC-2) and A-trisaccharide. (**C**) Close-up of the two fucose moieties (FUC-1 and FUC-2) binding on the GX P domain. Binding interactions were analyzed using PyMOL (version 2.1) with hydrogen bond interaction distances less than ~3.2 Å. The fucose molecule was held by direct hydrogen bonds with the side chains of E332, H334, E349, and T433 on one P domain monomer. (**D**) Close-up of the A-trisaccharide showing the binding interactions with the GII.27 P domain, where the blue spheres represent water molecules. The A-trisaccharide was held by direct hydrogen bonds with the side chains of D384 and R355, and the main chain of N354 on one monomer, while the other monomer held the A-trisaccharide with the side chain of K444 and main chain and G446. A hydrophobic interaction by Y447 and several water-mediated interactions were also observed (D383 and K444).

The GX and GII.27 P domains were prepared (6), co-crystallized with fucose and a panel of HBGAs (7, 8), and X-ray diffraction was processed (6) (Table 1). For GX, we could only observe two fucose molecules bound to the P domain dimer (Fig. 1A), despite screening numerous P domain-HBGA complex crystals at high resolutions. In the case of GII.27, we obtained X-ray data showing that a single A-trisaccharide molecule bound to the P domain dimer (Fig. 1A), as previously observed with other GII genotypes (7, 8). Complex crystals for the GII.27 P domain with other HBGA types were not formed. The electron density of the bound fucose and A-trisaccharide molecules was clearly discernible and modeled during the final refinement (Fig. 1B). The GX P domain held the fucose molecules with six direct hydrogen bonds, contributed by one P domain monomer (Fig. 1C). The GII.27 P domain and A-trisaccharide binding interaction was mediated by a network of hydrogen bonds, a hydrophobic interaction, and several water-mediated interactions by both P domain monomers (Fig. 1D), which was observed with other GII genotypes (7–10).

**TABLE 1:** Data collection and refinement statistics of P domain X-ray crystal structures

	GX fucose	GII.27 A-trisaccharide
Data collection		
Space group	P 32 2 1	P 1 21 1
Cell dimensions		
*a*, *b*, *c* (Å)	67.5 67.5 238.4	50.9 90.7 66.8
*α, β, γ* (°)	90.0 90.0 120.0	90.0 106.0 90.0
Resolution range (Å)	47.68–1.55 (1.58–1.55)	45.45–1.76 (1.80–1.76)
No. of unique reflections	91,925 (4,332)	57,076 (3,058)
*R*_merge_*^a^*	0.065 (1.417)	0.060 (0.533)
*R*_meas_*^b^*	0.068 (1.477)	0.064 (0.568)
*R*_pim_*^c^*	0.019 (0.407)	0.022 (0.194)
<I/σ(I)>	16.5 (1.4)	16.8 (2.8)
CC_1/2_	1.000 (0.643)	1.000 (0.910)
Completeness	99.8 (95.3)	99.0 (94.9)
Multiplicity	13.3 (12.5)	8.2 (8.1)
Refinement		
Resolution range (Å)	36.96–1.55 (1.59–1.55)	45.45–1.76 (1.80–1.76)
*R*_work_*^d^*	0.1580 (0.3043)	0.1486 (0.2086)
*R*_free_*^d^*	0.1937 (0.3645)	0.1964 (0.2580)
No. of atoms	5,540	5,680
Protein	4,726	4,913
Water	792	708
Ligand	22	59
B-factors (Å^2^)	29.98	26.57
Protein	28.06	25.41
Water	41.07	34.47
Ligand	43.60	28.04
RMS bond length (Å)	0.014	0.008
RMS bond angle (°)	1.253	0.945
Ramachandran plot statistics^*e*^		
Residues	620	634
Most favored region	97.63	95.77
Allowed region	2.20	4.07
Disallowed region	0.17	0.16
Clashscore	4.24	3.28
PDB ID	9OLU	9OLT
Mother solution	1.6 M magnesium sulfate heptahydrate, 0.1 M MES (pH 6.5)	0.17 M ammonium sulfate, 25.5% (wt/vol) PEG 4000

^*a*^ *R*_merge_ = Σh Σi |*I*(h)i – *I*h| / Σ*h* Σ*i I*(h)i where *I*h is the averaged intensity of all reflections *h*.

^*b*^ *R*_meas_ = Σh [N/ (*N *– 1)]^1/2^ Σi| *I*(ih) – *I*h |/Σh Σi *I*(ih).

^*c*^ *R*_pim_ = Σh [1/ (*N* – 1)]^1/2^ Σi| *I*(ih) – *I*h |/Σh Σi I(ih).

^*d*^ *R*_work_ and *R*_free_ = ∑|*F*obs – *F*calc| / ∑|*F*obs| × 100 for 95% of recorded data (*R*_work_) or 5% data (*R*_free_).

^*e*^ Determined using MolProbity.

**Fig 2:**
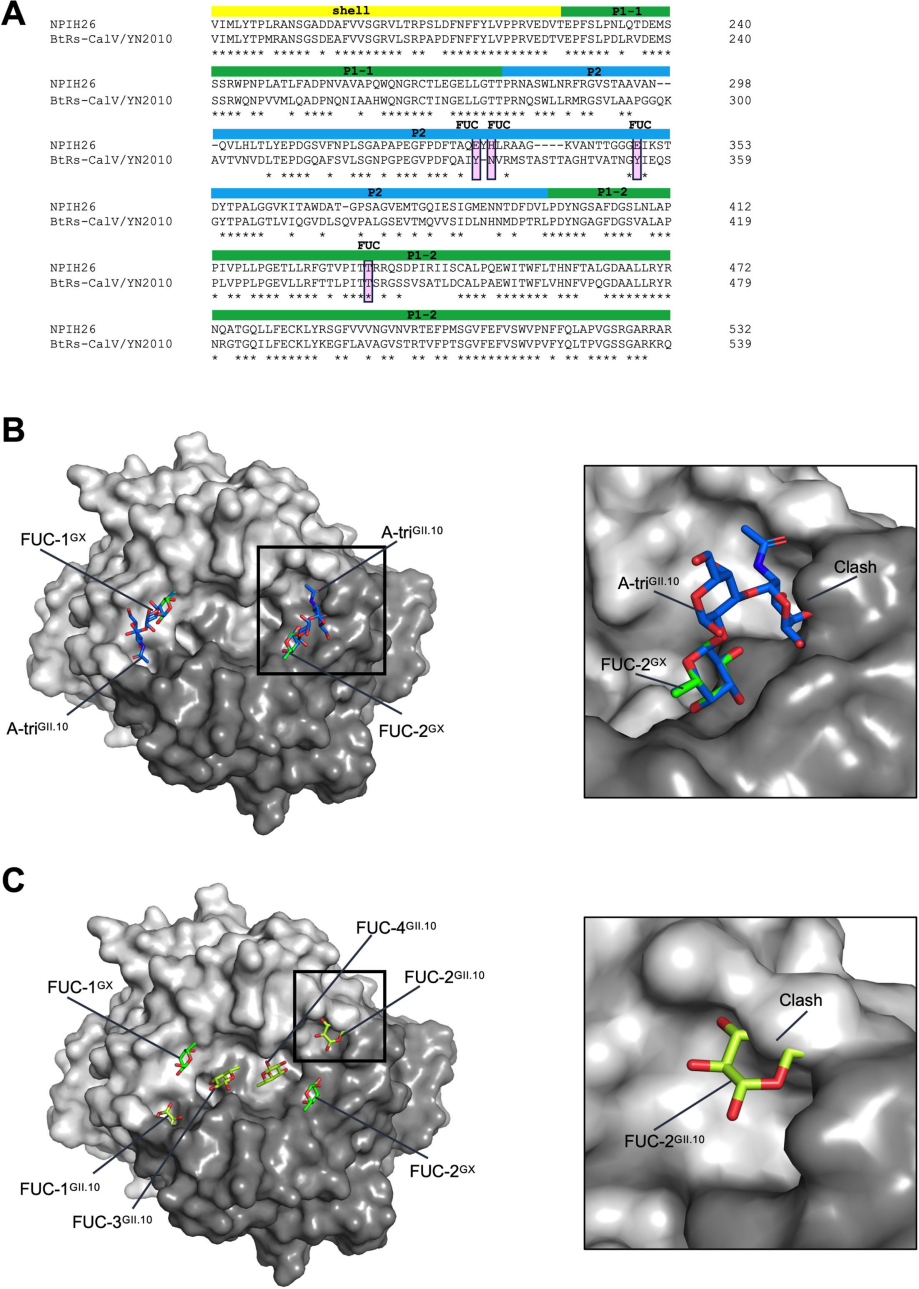
Comparisons of the GX fucose binding site with another bat and human norovirus. (**A**) A partial capsid amino acid sequence alignment of two bat norovirus NPIH26 and BtRs-CalV/YN2010 (KJ790198). The NPIH26 fucose binding residues are highlighted pink. Only one fucose binding residue was conserved in both NPIH26 (T433) and BtRs-CalV/YN2010 (T440). (**B**) The fucose moiety from the GII.10 P domain A-trisaccharide (A-tri^GII.10^) complex (3PA1) was superimposed onto the two fucose molecules (FUC-1/2^GX^) of the GX P domain fucose complex to demonstrate how an HBGA might bind on the GX P domain surface. The close-up (boxed region) shows that the terminal A-tri^GII.10^ moiety (*N*-acetyl-α-D-galactosamine) clashes with the GX P domain, which would likely obstruct HBGA binding at this site. (**C**) The GII.10 P domain can bind four fucose molecules (termed FUC-1/2/3/4^GII.10^, 4Z4R), where FUC-1/2^GII.10^ represents the typical GII HBGA binding pocket. These four fucose molecules were superimposed on the GX P domain, and the close-up (boxed region) shows the FUC-2^GII.10^ clashes on the GX P domain, indicating that this site is unlikely to bind fucose or HBGAs.

The X-ray screening data revealed that the GX P domain did not bind HBGAs, whereas in another ELISA-based study, a different bat norovirus (BtRs-CalV/YN2010) was found to bind to a panel of HBGA types (1). The two bat capsids shared 67.8% amino acid identity, and only one NPIH26-fucose binding residue was shared with BtRs-CalV (Fig. 2A). To better understand the distinct fucose and HBGA binding sites among GX and human GII noroviruses, we superimposed A-trisaccharide molecules onto the GX P domain fucose binding sites (Fig. 2B). In this model, the A-trisaccharide molecules clashed with the GX P domain in one monomer and were sterically close on the other monomer. We also superimposed fucose molecules from a GII.10 P domain fucose complex structure onto the GX P domain (Fig. 2C). In this model, the human norovirus fucose molecules clashed on the GX P domain, which was also the common GII HBGA binding site (7–10).

Overall, NPIH26 norovirus exhibited a divergent fucose binding site from human norovirus and had structural constraints for binding HBGAs, whereas the GII.27 norovirus bound an A-trisaccharide at the common GII HBGA pocket. Deciphering these interactions is important for understanding possible norovirus cross-species transmission mechanisms and therapeutic development strategies, respectively.

## Data Availability

Coordinates and structure factors were deposited into the Protein Data Bank under the following ID numbers: GX/NPIH26 bat norovirus protruding domain in complex with L-fucose, PDB ID 9OLU; GII.27 norovirus protruding domain complexed with A-trisaccharide, 9OLT.
